# Exploring changes in dietary intake, physical activity and body weight during chemotherapy in women with breast cancer: A Mixed‐Methods Study

**DOI:** 10.1111/jhn.12843

**Published:** 2021-01-07

**Authors:** Anja JThCM de Kruif, Marjan J. Westerman, Renate M. Winkels, Marije S. Koster, Irene M. van der Staaij, Maaike M. G. A. van den Berg, Jeanne H. M. de Vries, Michiel R. de Boer, Ellen Kampman, Marjolein Visser

**Affiliations:** ^1^ Department of Health Sciences Faculty of Science The Amsterdam Public Health Institute Vrije Universiteit Amsterdam Amsterdam The Netherlands; ^2^ Department of Epidemiology and Biostatistics Amsterdam UMC Location VUmc Amsterdam The Netherlands; ^3^ Division of Human Nutrition and Health Wageningen University Wageningen The Netherlands; ^4^ Department of Quality Assurance and Process Management Student & Educational Affairs Vrije Universiteit Amsterdam Amsterdam The Netherlands

**Keywords:** body weight, breast cancer, dietary intake, mixed methods, perceptions, physical activity

## Abstract

**Background:**

The present study aimed (i) to assess changes in dietary intake (DI), physical activity (PA) and body weight (BW) in breast cancer patients during chemotherapy; (ii) to describe how women explained, experienced and dealt with these potential changes; and (iii) to eventually develop lifestyle intervention strategies tailored to the women's personal needs during chemotherapy.

**Methods:**

A longitudinal parallel mixed‐method design was used with quantitative assessment of changes in dietary intake (24‐h recall, Appetite, Hunger, Sensory Perception questionnaire), physical activity (Short Questionnaire to Assess Health‐enhancing physical activity, Multidimensional Fatigue Inventory) and BW (dual‐energy X‐ray absorptiometry), in addition to qualitative interviews with 25 women about these potential changes during chemotherapy.

**Results:**

Most women who perceived eating less healthily with low energy intake (EI) and being less active before diagnosis continued to do so during chemotherapy, according to quantitative measurements. They struggled to maintain sufficient energy intake. Despite a lower than average reported EI, they unexpectedly gained weight and explained that fatigue made them even more inactive during chemotherapy. Active women usually managed to stay active because exercise was very important to them and made them feel good, although they also suffered from the side‐effects of chemotherapy. They found more ways to deal with taste, smell and appetite problems than women with a lower energy intake.

**Conclusions:**

The combination of the quantitative and qualitative data provided more insight into the changes in dietary intake, physical activity and BW during chemotherapy. The women's explanations showed why some women remain active and others need support to deal with changes in lifestyle factors such as healthy nutrition and fatigue.

## Graphical abstract

In this longitudinal mixed‐method study, we quantitatively assessed changes in diet, physical activity and body weight, and qualitatively assessed how individual women with breast cancer experienced, explained, and responded to these changes during chemotherapy, enriching and clarifying the quantitative data. The findings showed that some women remained active with fewer dietary problems, while others struggled with adequate food intake, opportunities to be active, and experienced unexpected weight gain. Women with breast cancer need support to be active and to cope with fatigue, and they need help with their eating habits to develop lifestyle intervention strategies tailored to their personal needs during chemotherapy.

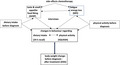

## INTRODUCTION

For breast cancer patients, the side‐effects of chemotherapy include unfavourable changes in body composition (i.e. increase in fat mass and loss of muscle mass) and weight changes.^1^ A meta‐analysis shows that body weight (BW) during chemotherapy increases with a mean of 2.7 kg (95% confidence interval = 2.0–3.3).^1^ These changes may have a profound negative influence on quality of life and self‐esteem in breast cancer survivors, and may increase the risk of several co‐morbidities, such as cardiovascular disease, diabetes mellitus and breast cancer recurrence.^2^, ^3^

Changes in lifestyle factors such as dietary intake (DI) and physical activity (PA) may influence changes in BW and body composition during chemotherapy.^4^, ^5^, ^6^, ^7^ DI during chemotherapy may be influenced by increased appetite and intake of energy‐dense comfort foods, which were found to be more common among women who gained weight during treatment.^8^ A decline in subjective taste perception and appetite is associated with weight loss.^5^, ^9^, ^10^ Lower intake of foods and drinks may be the result of experiencing a dry mouth, nausea, difficulty chewing, lack of energy as a result of fatigue and lower taste and smell perception.^11^ An earlier study reported that DI in patients with breast cancer just before start of chemotherapy was similar to a comparison group of women without breast cancer (2070 kcal day^–1^). However, during chemotherapy, the average EI of patients was 214 kcal day^–1^ lower than in the comparison group.^12^

When breast cancer patients become more physically active during therapy (e.g. because of a training intervention), they experience increased wellbeing, restored energy levels and a sense of purpose and control over their disease.^13^ However, the side effects of the treatment often compel women to reduce their daily activities.^14^ Their decision to reduce PA is often a result of fatigue, the need to conserve energy,^15^ difficulty staying focused because of ‘chemo brain’ (cancer‐therapy‐associated cognitive change),^16^ fear, possible injury, lack of time as a result of taking care of children and lack of motivation.^15^

Patients reported unanticipated weight gain during chemotherapy as a major concern. They experienced gaps in information on weight management and needed better information on dietary and lifestyle changes during and after chemotherapy, so that life can continue as normally as possible during this period.^5^, ^17^, ^18^ More knowledge about how individual women experience and respond to quantitative changes in DI, PA and BW can enrich and clarify evidence from quantitative measurements.^19^ This knowledge can be used to develop lifestyle intervention strategies and advice, tailored to the women's personal needs during chemotherapy.

The aim of this mixed‐methods study was the quantitative assessment of changes in dietary intake, physical activity and BW in breast cancer patients during chemotherapy, in addition to a qualitative description how women explain, experience and deal with these potential changes.

## MATERIALS AND METHODS

### Design

**FIGURE 1:**
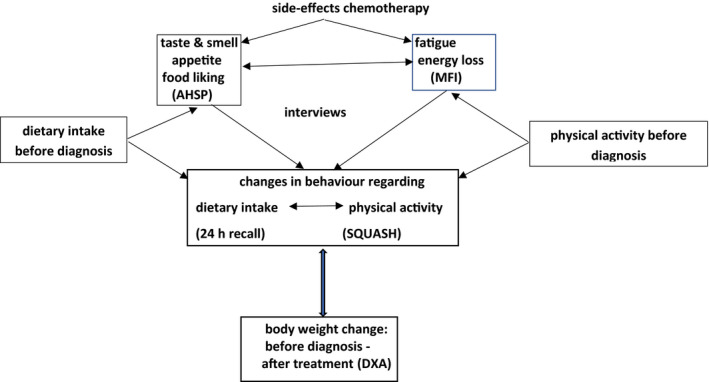
Conceptual behavioural model of women with breast cancer treated with chemotherapy and possible changes in dietary intake, physical activity and body weight. AHSP, Appetite Hunger Feelings and Sensory Perception questionnaire; SQUASH, Short Questionnaire to Assess Health‐enhancing physical activity; MFI, Multidimensional Fatigue Inventory; DXA, dual‐energy X‐ray absorptiometry

This study is part of the COBRA study (Change Of Body composition in BReast cancer: All‐in assessment).^19^, ^20^ We conducted a mixed‐methods study among breast cancer patients, using semi‐structured interviews, questionnaires and measurements on DI, PA and BW (Figure 1).

We expected the perspectives of patients in the qualitative part to complement the quantitative part, which is based on validated questionnaires and other quantitative measurements.^21^ In addition, the approach was enriched by the use of triangulation to enhance the reliability and credibility of the findings.^22^, ^23^ We did not aim to make generalising statements about an entire population, but rather contextual (i.e. within this group of 25 women) statements that reflect the spectrum of individual experiences related to the objective individual measurements.^24^

Participants were contacted for measurements and in‐depth interviews at three time points (Table 1): at enrollment into the study after diagnosis, before the start of chemotherapy (T1); between the third and fourth cycle of chemotherapy (T2); and 6 weeks after the last cycle of chemotherapy (T3).

**TABLE 1:** Overview of timing and type of quantitative and qualitative measurements among 25 women with breast cancer

*n* = 25	T1 after diagnosis, pre chemotherapy	T2 mid‐way chemotherapy	T3 6 weeks post chemotherapy
Quantitative measurements			
Taste, smell and appetite^a^	x	X^f^	x
Energy intake^b^		x^g^	
Physical activity^c^	x		x
Fatigue ^d^	x		x
Body weight^e^	x		x
Qualitative measurements			
Self‐reported body weight		x	
Perceptions of women on potential changes in dietary intake, physical activity and body weight^h^	x	x	x

^a^ AHSP: Appetite Hunger Feelings and Sensory Perception questionnaire.

^b^ 24‐h dietary recall.

^c^ SQUASH: Dutch Short Questionnaire to Assess Health‐enhancing physical activity.

^d^ MFI: Multidimensional Fatigue Inventory.

^e^ DXA: dual‐energy X‐ray absorptiometry.

^f^ Assessed on same days as 24‐h dietary recalls.

^g^ Based on two 24‐h dietary recalls on randomly chosen days during chemotherapy.

^h^ Interviews.

The Medical Ethics Committee of Wageningen University approved the COBRA study (ABR NL40666.081.12). All procedures performed in studies involving human participants were conducted in accordance with the ethical standards of the institutional and/or national research committee and with the 1964 Declaration of Helsinki and its later amendments or comparable ethical standards. Informed consent was obtained from all individual participants included in the study.

### Study sample

We selected 25 participants from the total COBRA‐study cohort (*n* = 181). All women underwent surgery and chemotherapy for breast cancer, in variable order. Via purposive sampling,^25^, ^26^ we selected a heterogeneous group of respondents representing the following characteristics: age (young versus older), type of chemotherapy (adjuvant and neo‐adjuvant), body mass index (BMI) (>25 and <25 kg m^–2^), menopausal status (pre and post), and patients treated in different hospitals.

### Data collection

#### Quantitative data collection

From the Appetite, Hunger, Sensory Perception (AHSP) questionnaire,^27^ a 29‐question self‐assessment tool answered on a five‐point Likert scale (Table 1), we used three categories: taste (eight items, score range 8–40), smell (six items, range 6–30) and appetite (six items, range 6–30). Higher scores indicate a more positive self‐judgement on taste, smell and appetite. Trained dietitians conducted two 24‐h dietary recalls by telephone on randomly chosen days at T2, using a standardised protocol. The intake of total energy (kcal) was calculated in the computation module of Compl‐eat™ using the Dutch Food Composition Table 2013.^28^ BW was assessed with a dual‐energy X‐ray absorptiometry (DXA) scan at T1 and T3. The Short Questionnaire to Assess Health‐enhancing physical activity (SQUASH) was used to assess PA.^29^ Reported activities were subdivided into three intensity categories (min day^–1^): light [1.6–2.9 metabolic equivalent of tasks (MET)], moderate (3–5.9 METs), vigorous (≥6 METs). One MET is the energy expenditure at rest. We then calculated adherence to the 2017 Dutch Physical Activity guideline,^30^ which recommends a minimum of 150 min of moderate to vigorous activity per week. The Multidimensional Fatigue Inventory (MFI‐20) is a self‐reporting tool consisting of twenty propositions on five dimensions of fatigue (general fatigue, physical fatigue, reduction in activity, reduction in motivation and cognitive fatigue) and its consequences.^31^, ^32^, ^33^

#### Qualitative data collection

The semi‐structured interviews were based on an interview guide that included topics such as side‐effects of chemotherapy (focus on smell and taste), experiences and explanations of changes in DI, PA and BW, as well as perceptions on dealing with these changes.^26^ The interviews were conducted at the participant's home. Oral permission for audiotaping was obtained before each interview.

### Data analysis

#### Quantitative data

We first dichotomised the women based on EI at T2, PA and BW at baseline (T1) and T3. All intake parameters were complete for 117 participants of the total COBRA population (*n* = 181).

We compared EI during chemotherapy (T2) with the mean (SD) EI of 1779 (55.7) kcal day^–1^ of the total COBRA population of *n* = 117 persons during chemotherapy (12) and created two groups: above (high) or below (low) that averaged energy intake. For PA at T1 and T3, we applied the Dutch Physical Activity Guideline^30^; performance below the guideline at T3 compared to T1 was classified as inactive and above the guideline at T3 compared to T1 was classified as active. For changes in BW, we calculated the changes in kg between T1 and T3 measured by DXA.

In addition, we compared taste, smell and appetite with the mean scores of the total COBRA population^12^: for taste [mean (SD) 22.0 (0.57)], smell [mean (SD) 20.6 (0.42)] and appetite [mean (SD) 18.7 (0.50)]; above (high) or below (low) mean scores. For fatigue at T1 and T3, we calculated the MFI, and compared the values with the mean scores of a reference group of cancer survivors; above (high) or below (low) the mean scores. BMI was classified such that <25 kg m^–2^ was considered low and BMI ≥ 25 kg m^–2^ was considered high.

#### Qualitative data

All audiotaped interviews were transcribed verbatim and a synopsis was written for each respondent. To identify, analyse and describe patterns in respondents’ individual experiences, athematic analysis^34^, ^35^ was carried out during which, after close reading and coding of the transcribed interviews, we formulated subthemes and eventually overarching themes. Interview data were managed with maxqda (VERBI Software, Marburg, Germany.^36^ All subthemes and themes were described in a mind map: one for each individual. In this way, we identified the essence of each theme, searched for relations through constant comparison across cases, looked for deviant cases, and analysed variation within and between cases.

#### Combining quantitative and qualitative data

All mind maps included individual results of the quantitative measurements (AHSP, BW, 24 h dietary recall, SQUASH and MFI) to find characteristics that further differentiate the themes. Subsequently, we gathered the relevant (sub)themes and quantitative measurements necessary to answer the research question. Finally, all the data were fit into the overarching story emerging from the data, aiming to gain insight into differences and similarities between the women regarding the findings from the quantitative and qualitative analyses.

## RESULTS

Twenty‐five women from the COBRA study were included in the present study (Table 2).

**TABLE 2:** Baseline characteristics of the participants

Characteristics	*n* = 25
Age in years, mean (range)	50.4 (25–67)
Body mass index (kg m^–2^) at T1 pre‐chemotherapy, mean (range)	Mean 24.9 (19.3−32.4)
Menopausal status at T1, *n* (%)^a^	
Premenopausal	10 (40%)
Perimenopausal	3 (12%)
Postmenopausal	12 (48%)
Treatment chemotherapy, *n* (%)	
Adjuvant	17 (68%)
Neoadjuvant	8 (32%)
Type of chemotherapy, *n* (%)^b^	
FEC/docetaxel	9 (36%)
TAC	14 (56%)
ACPT^c^	2 (8%)
Received radiotherapy, *n* (%)	18 (72%)
Received hormonal therapy, *n* (%)	22 (88%)

^a^ Patients with uterine extirpation (*n* = 3).

^b^ FEC: F – fluorouracil (5 FU), E – epirubicin, C – cyclophosphamide. Docetaxel: also called Taxotere (Sanofi Mature IP, Paris, France). TAC: T – docetaxel (also called Taxotere), A – doxorubicin (also called Adriamycin), C – cyclophosphamide. ACPT: A – adriamycin (doxorubicin), C – cyclophosphamide P = paclitaxel, T = docetaxel.

^c^ These patients also received immunotherapy (trastuzumab) in combination with chemotherapy.

### Quantitative results

#### Dietary intake

The average reported EI of the 25 women during chemotherapy of 1761 kcal (range 1182–3102 kcal) was similar to the average reported EI of all women in the total COBRA study of 1779 (55.7) kcal.^12^ Women (*n* = 11) with an EI above average (mean 2165 kcal; range 1783–3102 kcal) were almost equally distributed between a lower BMI (*n* = 6) and a higher BMI (*n* = 5), just like women (*n* = 14) with an EI below average (mean 1442 kcal; range 1182–1738 kcal) were equally distributed between a higher BMI (*n* = 7) and a lower BMI (*n* = 7). Women with an EI above average were approximately as active (60% versus 54% met PA recommendation) and as fatigued (70% versus 77%) as women with an EI below average during chemotherapy. Those with a higher EI less often gained BW (64% versus 79%). Scores on appetite, taste and smell were low for most women, regardless of their EI. Women with lower EI during chemotherapy more often had a lower taste score (91%) than women with a higher EI (70%) (Table 3).

**TABLE 3:** Assessment of changes in dietary intake, physical activity and body weight during chemotherapy

				Energy intake^a^ (kcal)			Physical activity^b^ (min week^–1^) T3^f^				Body weight^d^ difference (kg) T1–T3	
				≥1780 *n* = 11	<1780 *n* = 14		≥150 *n* = 13		˂150 *n* = 10		Increase *n* = 18	Decrease *n* = 7
Energy intake^a^ (kcal)	≥1780 *n* = 11					6 (46%)		4 (40%)		7 (39%)		4 (57%)
	<1780 *n* = 14					7 (54%)		6 (60%)		11 (61%)		3 (43%)
Physical activity^b^ (min week^–1^) T3^f^	≥150 *n* = 13		6 (60%)	7 (54%)						8 (47%)		5 (83%)
	˂150 *n* = 10		4 (40%)	6 (46%)						9 (53%)		1 (17%)
Body weight^d^ difference (kg) T1–T3	Increase *n* = 18		7 (64%)	11(79%)		8 (62%)		9 (90%)				
	Decrease *n* = 7		4 (36%)	3 (21%)		5 (38%)		1 (10%)				
BMI T1	≥25 kg m^–2^ *n* = 12		5 (45%)	7 (50%)		4 (31%)		7 (70%)		7 (39%)		5 (71%)
	˂25 kg m^–2^ *n* = 13		6 (55%)	7 (50%)		9 (69%)		3 (30%)		11 (61%)		2 (29%)
Fatigue^c^ T3^f^	High *n* = 17		7 (70%)	10 (77%)		7 (54%)		10 (100%)		13 (76%)		4 (67%)
	Low *n* = 6		3 (30%)	3 (23%)		6 (46%)		‐		4 (24%)		2 (33%)
Appetite^e^ (6–30) T2^f^	Normal/high *n* = 5		2 (20%)	3 (25%)		5 (42%)		‐		4 (24%)		1 (20%)
	Low *n* = 17		8 (80%)	9 (75%)		7 (58%)		10 (100%)		13 (76%)		4 (80%)
Taste^e^ (8–40) T2^f^	Normal/high *n* = 4		3 (30%)	1 (9%)		2 (17%)		2 (22%)		3 (19%)		1 (20%)
	Low n = 17		7 (70%)	10 (91%)		10 (83%)		7 (78%)		13 (82%)		4 (80%)
Smell^e^ (6–30) T2^f^	Normal/high *n* = 2		1 (10%)	1 (8%)		2 (100%)		‐		2 (12%)		‐
	Low *n* = 20		9 (90%)	11 (92%)		10 (50%)		10 (100%)		15 (88%)		5 (100%)

^a^ 24‐h dietary recall, above or below mean 1779 kcal ± 55.7 kcal in the total COBRA population for patients with breast cancer.

^b^ Short Questionnaire to Assess Health‐enhancing physical activity above or below 150 min at least moderately intense physical activity.

^c^ Multidimensional Fatigue Inventory: above or under below mean scores of reference group of cancer survivors.

^d^ Dual‐energy X‐ray absorptiometry scan, difference body weight (kg) T1–T3.

^e^ Appetite Hunger Feelings and Sensory Perception questionnaire: above or below mean scores of total Cobra population (mean appetite 18.7; mean taste 22.0; mean smell 23.3). Higher scores indicate more positive self‐judgement on the questionnaire categories.

^f^ Missing values: for two women on physical activity and fatigue, as well as taste, smell and appetite.

#### Physical activity

All women were moderately intensely physically active for ≥150 min per week at T1. At T3, approximately half of the women (13/23) were physically active for more than 150 min per week. Active women during chemotherapy (women who were active at T1 and stayed active at T3) (*n* = 13) more often had a lower BMI (69%) before treatment than inactive women (women who were active at T1 and below guideline PA at T3) (*n* = 10) (30%). They were more likely to have an EI above average (60% vs. 40%). The majority gained BW (62%), although inactive women were more likely to gain BW (90%). Active women during chemotherapy were less fatigued (54% versus 100%) and less often showed appetite problems (100% versus 58%) and smell problems (100% versus 50%) compared to inactive women (Table 3).

#### Body weight

Women had a mean baseline (T1) BMI of 24.9 kg m^–2^ (19.3–32.4). Their average BW at T1 was 71.4 kg (range 54.0–93.1 kg), the average difference in BW between T1 and T3 was 0.8 kg (range −6.5 to +4.5 kg). The majority of women (72%, *n* = 18) showed an increase in BW between T1 and T3: mean +1.9 kg (range 0.1–4.5); 28% (*n* = 7) showed a decrease in BW: mean −2.1 kg (range –0.2 to −6.5 kg). Women who gained weight were less likely to have an EI above average (39%) than women who lost weight (57%). Women with weight gain were less likely to meet the PA recommendation during chemotherapy (47% vs. 83%) and more often had a low BMI at baseline (61% vs. 29%). Women who gained weight and women who lost weight both showed high levels of fatigue (76% vs. 67%), appetite problems (76% vs. 80%) and taste problems (82% vs. 80%). Women with weight gain reported less smell problems (88% vs. 100%) than women with weight loss (Table 3).

### Qualitative results

#### Dietary intake

Most women with an EI above average reported they perceived their diet was healthy before diagnosis and were able to maintain their diet despite chemotherapy‐related complaints (Table 4). Most women with low EI indicated they did not always eat healthily and were less aware of what healthy eating was before the diagnosis. These women continued to struggle during chemotherapy. Sometimes eating was such a problem that they were happy when they could eat at all, healthily or not:

… happy when it went down, no matter what it was. Preferred sausage rolls to bread, baked potatoes and fries were tasty, no more fight against candy. [woman with low EI, BMI high, PA low, fatigue high, BW increased, Taste (T), Smell (S) and Appetite (A) low]

**TABLE 4:** Overview of the most important experiences during chemotherapy, according to women's dietary intake, physical activity and change in body weight

	Before chemotherapy	Changes during chemotherapy	Explaining changes	Dealing with changes
Dietary intake				
Above average	Quite healthy diet BMI at T1: equally distributed between lower (≤25 kg m^–2^) and higher (>25 kg m^–2^)	Able to maintain their diet despite chemotherapy‐related complaints, and appetite, taste and smell problems Gained body weight	Suffering from side‐effects chemotherapy, but a healthy diet is very important Chemotherapy is temporary, just keep eating as normally as possible	Functional eating and more, smaller portions Easy‐to‐manage healthy diet taking less effort to prepare Use of dietary supplements
Below average	Less healthy BMI at T1: equally distributed between lower and higher BMI	Intake: quantity takes precedence over healthy choices Insufficient energy intake results in hospital admissions Gain more body weight More intake problems during Docetaxel More problems with taste and structure of food	No clear expectations/no preparation for side‐effects of chemotherapy Advice on a ‘normal diet’ not in line with desire to do more themselves with healthy food and supplements. Need for more information.	Functional eating Improvement of eating habits with less success than women with intake above average.
Physical activity				
According to guideline	Active BMI at T1 often lower	Able to remain active Gained body weight	Valued being active greatly and tried to keep active as much as possible	Struggled hard to maintain activity level for health reasons despite fatigue Adjusting their pace Made choices to spend time on physical activity, not on increasing the number of hours of work OR Found it easy to remain active because limited experienced side‐effects of chemotherapy
Below guideline	Inactive BMI at T1 often higher	Felt overwhelmed by fatigue and often unable to be active Gained more body weight	Never liked to be active AND/OR Too tired (fatigue, neuropathy, bone pain)	Overwhelmed by fatigue and experienced no possibilities to change
Body weight				
Gain	BMI at T1 lower	Unexpected weight gain Some gained less weight by intentionally eating less but remained inactive	Desire to lose weight Constant good appetite, difficult to stop eating	Being less active Intake mainly tasty (high‐energy) foods Could not stop eating
Loss	BMI at T1 higher	Great difficulty eating Do anything not to lose weight Sometimes happy to lose weight	Focus more on diet than on being physically active	Could not eat enough to avoid weight loss OR Intentional weight loss

Abbreviations: BMI, body mass index; T1, before the start of chemotherapy.

Some women struggled because of extremely bad taste, especially during the last three courses with Docetaxel, and could not eat at all.

Some women with higher intake were matter‐of‐fact about their eating problems:

The chemo is temporary, I wasn't looking for a solution; just keep eating as normally as possible, even if it's not tasty … [woman with high EI, BMI high, PA not available (NA), fatigue NA, BW decreased, T, S, A: NA]

Women with lower intake indicated that they first needed to experience what it was like to be treated with chemotherapy. They found it difficult when health care professionals (HCPs) recommended ‘a normal diet’ during chemotherapy, because they assumed that a normal diet was not good enough and they longed to do more:

I was searching for supplements, vitamins yes or no, no grapefruit, but orange? What is or isn't healthy to eat now? (woman with low EI, BMI high, PA low, fatigue high, BW increased, T, S and A low)

Women with higher EI looked for creative solutions to be able to continue their intake, such as adding herbs, ketchup, salt for better taste; sucking ice cubes to avoid a metallic taste; eating yoghurt instead of bread; eating fruits such as grated apple because it went down easier. They mainly cooked easy‐to‐prepare meals, such as oatmeal in the morning and ready‐to‐eat meals in the evening. They more often ate smaller portions throughout the day. Some women reported that they perceived eating more healthily than before diagnosis and added various healthy alternatives and supplements to their diet.

Women with lower EI also tried to change their eating habits to accommodate a damaged and dry mouth, no appetite and loss of taste, although their aversion to almost every food was hard to overcome. They continued to struggle because they realised that it was necessary and called it functional eating:

I eat because I know I have to, but it's not going well and I certainly don't enjoy it (woman with low EI, BMI high, PA low, fatigue high, BW increased, T, S and A low)

### Physical activity

Some women meeting the PA recommendation during chemotherapy who engaged in intensive sports before diagnosis tried to maintain this as much as possible during chemotherapy, although it was not always easy:

Yesterday I walked 20 kilometres, I really like that […] But it took me half an hour longer because I had diarrhoea every two hours. There I was, sitting with my roll of toilet paper and body cream, with my head in the bushes … (woman with PA high, BMI low, EI normal, fatigue high, BW increased, T and S low, and A normal)

They explained that keeping fit was very important to them:

I need to keep in shape; I could have worked a bit more but then I couldn't go cycling; cycling just takes precedence … (woman with PA high, BMI low, EI normal, fatigue low, BW decreased, T, S and A low)

Other women remained physically active mainly because the hospital offered exercise training for cancer patients. This was mainly experienced as meaningful contact with fellow patients aimed at recognition and sharing, not as PA.

Although active women were also tired, they did not want to give into it. They tried to keep cycling and walking as much as possible, they continued to work, but for fewer hours and they adjusted their pace (e.g. in household activities).

Most women who were inactive during chemotherapy indicated that before diagnosis PA was not their first priority and they usually did not like it. During chemotherapy, they experienced side‐effects such as neuropathy and bone pain and their fatigue prevented activity:

Well, when I was very tired, I got up in the morning … Hoping the day would be over as soon as possible … so I could go back to bed … I just didn't know how to get through the day … (woman with PA low, BMI high, EI low, fatigue high, BW increased, T, S and A low)

Some of them experienced even minimal PA as an almost insurmountable threshold and felt unable to change this:

At a certain point all your energy simply goes into learning to deal with the complaints you have (woman with PA low, BMI high, PA low, fatigue high, EI low, BW increased, T, S and A low)

Some hesitated joining activities in a group because of their hair loss and the deterioration of their breasts after surgery. They related their appearance to their female identity.

### Body weight

Most women noticed unexpected weight gain during chemotherapy:

I was not allowed to exercise during the last cycles of chemotherapy because of my lungs [radiation effect], so gradually another kilo is added (woman with weight gain, BMI high; EI low; PA normal, fatigue low, T and S low, A normal)

To their surprise, some women who were expecting weight gain based on information from HCPs, actually lost weight.

Some women attributed their weight gain mainly to eating tasty (high‐energy) foods, or not being able to stop eating. Others related it to decreased PA, not to changes in their diet.

Especially women with a lower BMI at diagnosis intentionally gained weight to be able to get through chemotherapy.

Other women, most of them with a higher BMI at diagnosis, were happy to lose some weight:

They said with this chemo you don't lose weight but you gain weight; I’ve lost three kilos, I'm happy … I felt I was 10 kilos too heavy (Woman with weight loss, BMI high, low EI, PA normal, fatigue high, A, T and S NA)

Most women focused on their diet, and how to deal with it, rather than on physical activity:

I did more with yoghurt and stuff and with fruit and vegetables, lettuce, I'd be able to control it a little more … I started fitness, stopped again … that's not my thing (woman with weight loss, BMI low, EI low, PA low, fatigue high, T and S low and A normal)

## DISCUSSION

In this mixed‐methods study, we aimed to assess changes in dietary intake, physical activity and BW in breast cancer patients during chemotherapy. We divided women into two groups for each lifestyle factor based on quantitative data: EI above and below the average, above and below the PA recommendation, and BW increase and decrease during chemotherapy. Through interviews, we determined how women experienced, explained and tried to deal with these changes. These results are complementary and explanatory to the quantitative measurements.

Our study is concordant with the recently published study by Da Costa Marinho *et al*.^37^ who observed a negative impact of chemotherapy on meal enjoyment and taste changes in breast cancer patients receiving chemotherapy. In addition, BW and BMI increased slightly, as described by Bernhardson *et al*.^38^ Almost all women in the present study experienced this negative impact independent of DI, PA or changes in BW. It appears that changes in DI, PA and BW also partly depend on how women deal with them.

We found that most women suffered taste (17/25), smell (20/25) and appetite (17/25) problems, regardless of their intake. The majority of interviewed women indicated having many problems with the taste and structure of food, and struggled to find solutions. All women aimed for sufficient DI through functional eating, which is synonymous with ‘eating for the sake of eating’ and in line with the studies of Bernhardson *et al*.^38^ and Kwok *et al*.^5^ It is not exactly clear why women with a higher EI were more successful at finding creative solutions to maintain their EI at sufficient levels than women with a lower intake. Women with a lower EI reported needing information about a healthy diet and whether or not to take supplements. Moreover, they needed information on alternatives for food intake to meet their dietary requirements.^39^

Although the majority of women were physically active during chemotherapy (15/25), only a few felt more fit during chemotherapy. The review by Abdin *et al*.^40^ suggests that PA interventions for women with breast cancer have positive results, such as feeling better. However, women in our study who participated in such a programme experienced it mainly as an opportunity for peer‐to‐peer contact and only secondly as an opportunity to be physically active. Especially, the inactive women indicated, in line with Henry *et al*.,^41^ that the impact of fatigue and neuropathy affected their ability to exercise. Women who were generally more active before chemotherapy greatly valued being active and tried to stay as active as possible, for example by adjusting their pace. Their baseline BMI was relatively lower and despite more taste problems they had a higher EI than inactive women, possibly because they were more creative in finding ways to continue eating. The level of PA during chemotherapy showed less fatigue (54%) for active women and more fatigue (100%) for inactive women. This is in line with the findings of Carayol *et al*.^42^ who assessed the effect of an exercise‐diet intervention during chemotherapy on cancer‐related fatigue. Fatigue was significantly improved by exercising, and BMI was significantly lower, although they found no significant effect on nutritional intake and physical activity.^43^

Some of the women were reluctant to continue their training routine because of visible side‐effects of chemotherapy, such as needing a wig because of hair loss. For them, this aspect was linked to their female identity. Courneya *et al*.^43^ and Browall *et al*.^44^ reported this aspect as one of the treatment‐related barriers to PA. The information need for PA was less clear, probably because PA was not deemed as important as their diet.

Women with weight gain had a lower EI during chemotherapy and were more inactive and tired than women who lost weight. By contrast to expectations, 61% of women with weight gain have an EI below average. Women themselves attributed their weight gain to being less active than usual. On average, women gained 0.8 kg BW between T1 and T3 during chemotherapy, similar to the weight change reported in the COBRA study.^20^ Before chemotherapy, a large part of the women focused on losing a few kilos, and half of them had a BMI ≥25 kg m^–2^ at the start of the study. In line with the studies by Klok *et al*.^5^ and de Kruif *et al*.,^45^ they experienced little support from HCPs to lose weight during chemotherapy. They experienced unintentional changes in BW as concerning, possibly because it undermined their feelings of control, as found in earlier studies.^5^ Compared to women with a baseline BMI ≥25 kg m^–2^ (7/12; 58%), women with BMI <25 kg m^–2^ (11/13; 85%) had an above average EI approximately as often and were more likely to be active, although they more often gained BW. On average, women with low BMI had a slightly higher intake (mean 1789 kcal, range 1182–3102 kcal) than women with high BMI (mean 1712 kcal, range 1289–2588 kcal).

### Strengths and limitations

The major strength of the present study is the mixed‐methods design. The combined results provide more insight into how women themselves explained and dealt with the changes in EI, PA and BW. Because this is the first study to undertake these types of analyses, the results can be considered a first step towards understanding similarities and differences between women associated with specific changes in BW and weight‐related lifestyle factors.

We selected the 25 women from the COBRA population by purposive sampling,^46^ based on characteristics that are important according to literature rather than on the COBRA population itself. As a result, these 25 women are expected to adequately reflect the general population of women with breast cancer treated with chemotherapy. That may explain why we found a lower score on appetite, taste and smell and a slightly lower score on EI than in the COBRA study.^12^

It is a well‐known problem that energy intake is difficult to assess. The limitation in our research is that we had no information about the basic energy needs of women. The energy needs of people are determined by their basal metabolic rate and physical exercise. We could report only on the latter. We therefore repeated the analyses on the basis of kcal kg^–1^ BW. Three women were classified differently, although this did not materially change the results. Also, misclassification of EI could affect the findings because people with a higher BMI are more prone to under‐reporting and people with a lower BMI are more prone to over‐reporting.^47^

We divided women into two groups for EI, PA and BW at baseline (T1) and T3: above or below the Dutch PA guideline and averages for EI, taste, smell and appetite of the COBRA population. To our knowledge, no general cut‐off points have been or can be determined for measurements of EI (EI depends on a woman's need), nor for taste, smell, appetite and fatigue for women with breast cancer aiming to dichotomise them into lower versus higher. The use of the guideline for PA and the mean scores of the entire COBRA cohort for EI, taste, smell and appetite and fatigue were necessarily arbitrary and may have influenced our results and conclusions.

Another limitation of the present study is our focus on physical lifestyle factors. In addition to these physical factors, psychological and social‐environmental factors, including socio‐economic status, education and knowledge of nutrition and lifestyle, are also expected to influence lifestyle and how women experience and deal with changes in BW and weight‐related lifestyle. We need further research into these factors to develop tailored dietary advice and PA strategies, which can be implemented to provide high‐quality health care to breast cancer patients during chemotherapy.

## CONCLUSIONS

The unique combination of quantitative and qualitative data has provided good insight into how women themselves explain and deal with the changes in dietary intake, physical activity and BW during chemotherapy. Women with a lower reported healthy intake and physical activity before diagnosis struggled more with sufficient and healthy dietary intake during chemotherapy than other women. They were overwhelmed by fatigue, barely saw possibilities of being active and experienced more unexpected weight gain. They need support to find ways to do more with respect to EI, PA and BW changes. To support them with respect to being active during chemotherapy, women with a low intake needed help especially with their eating habits, and inactive women needed help to deal with fatigue.

## CONFLICT OF INTEREST

The authors declare that they have no conflicts of interest.

## AUTHOR CONTRIBUTION

JK, MW, MdB and MV contributed to the conception and design of the study. JK, MB, RW and EK were responsible for the data collection. JK, MW, MK, IS, MdB, JV and MV were responsible for the analysis and interpretation of data. All authors contributed to the and to the writing and revision of the paper. All authors critically reviewed the manuscript and approved the final version submitted for publication.

## TRANSPARENCY DECLARATION

The authors affirm that this manuscript is an honest, accurate and transparent account of the study being reported. The authors affirm that no important aspects of the study have been omitted and that any discrepancies from the study as planned have been explained.
